# Amplification and Overexpression of Hsa-miR-30b, Hsa-miR-30d and *KHDRBS3* at 8q24.22-q24.23 in Medulloblastoma

**DOI:** 10.1371/journal.pone.0006159

**Published:** 2009-07-07

**Authors:** Yuan Lu, Sarra L. Ryan, David J. Elliott, Graham R. Bignell, P. Andrew Futreal, David W. Ellison, Simon Bailey, Steven C. Clifford

**Affiliations:** 1 Northern Institute for Cancer Research, Newcastle University, Newcastle upon Tyne, United Kingdom; 2 Institute of Human Genetics, Newcastle University, Newcastle upon Tyne, United Kingdom; 3 Cancer Genome Project, Wellcome Trust Sanger Institute, Wellcome Trust Genome Campus, Hinxton, Cambridge, United Kingdom; 4 Department of Pathology, St. Jude Children's Research Hospital, Memphis, Tennessee, United States of America; Roswell Park Cancer Institute, United States of America

## Abstract

**Background:**

Medulloblastoma is the most common malignant brain tumour of childhood. The identification of critical genes involved in its pathogenesis will be central to advances in our understanding of its molecular basis, and the development of improved therapeutic approaches.

**Methodology/Principal Findings:**

We performed a SNP-array based genome-wide copy number analysis in medulloblastoma cell lines, to identify regions of genomic amplification and homozygous deletion, which may harbour critical disease genes. A series of novel and established medulloblastoma defects were detected (*MYC* amplification (*n = 4*), 17q21.31 high-level gain (*n = 1*); 9p21.1–p21.3 (*n = 1*) and 6q23.1 (*n = 1*) homozygous deletion). Most notably, a novel recurrent region of genomic amplification at 8q24.22–q24.23 was identified (*n = 2*), and selected for further investigation. Additional analysis by interphase fluorescence *in situ* hybridisation (iFISH), PCR-based mapping and SNP-array revealed this novel amplification at 8q24.22–q24.23 is independent of *MYC* amplification at 8q24.21, and is unique to medulloblastoma in over 800 cancer cell lines assessed from different tumour types, suggesting it contains key genes specifically involved in medulloblastoma development. Detailed mapping identified a 3Mb common minimal region of amplification harbouring 3 coding genes (*ZFAT1, LOC286094, KHDRBS3*) and two genes encoding micro-RNAs (hsa-miR-30b, hsa-miR-30d). Of these, only expression of hsa-miR-30b, hsa-miR-30d and *KHDRBS3* correlated with copy number status, and all three of these transcripts also displayed evidence of elevated expression in sub-sets of primary medulloblastomas, measured relative to the normal cerebellum.

**Conclusions/Significance:**

These data implicate hsa-miR-30b, hsa-miR-30d and *KHDRBS3* as putative oncogenic target(s) of a novel recurrent medulloblastoma amplicon at 8q24.22–q24.23. Our findings suggest critical roles for these genes in medulloblastoma development, and further support the contribution of micro-RNA species to medulloblastoma pathogenesis.

## Introduction

Medulloblastoma is an invasive embryonal tumour of the cerebellum, and the most common malignant brain tumour in children. Although overall five-year survival rates of 60–70% are now achieved, a significant proportion of cases will die from their disease, and the intensive chemotherapeutic and radiotherapy regimes employed in treatment are associated with long-term neuroendocrine and cognitive dysfunction in surviving patients. Advances in our understanding of the biology of medulloblastoma will be essential to future improvements in therapeutic outcome, through strategies including the identification of specific targets for the development of novel therapies, and biomarkers for improved treatment stratification [1], [2].

The identification of specific genetic defects has been central to advances in our understanding of the molecular basis of medulloblastoma. A series of non-randomly mutated genes have been identified, which have, in turn, led to the characterisation of critical roles for their associated biological pathways in sub-sets of cases; the Wnt/Wingless (WNT) signalling pathway is activated by mechanisms including *CTNNB1* mutation in 10–15% cases, and the Sonic hedgehog (SHH) pathway in activated in a further 20–25%, predominantly by mutations in *PTCH1* [1]–[5]. The most prevalent genomic amplifications reported to date affect the *MYC* and *MYCN* oncogenes (each in 5–15% of cases), and homozygous deletions of established tumour suppressor genes, including *CDKN2A* and *P14ARF*, have been described [6]–[8]. Importantly, genetic defects have therapeutic relevance; markers of favourable (WNT activation) and poor (*MYC*/*MYCN* amplification) prognosis have been identified and validated in clinical trials cohorts [6], [9], [10], and small molecule inhibitors of the SHH pathway are under pre-clinical development [11].

Despite these advances, the critical genes involved in medulloblastoma development are otherwise poorly understood, and specific genetic defects remain to be identified in the majority of cases. We therefore undertook a comprehensive SNP-array based analysis of copy number defects in medulloblastoma cell lines, to identify regions of genomic amplification and homozygous deletion, which may harbour critical medulloblastoma genes. We report the validation, mapping and further characterisation of recurrent novel genetic regions identified, in medulloblastoma cell lines and primary tumours, and use these data to identify putative target gene(s) which may contribute to medulloblastoma development.

## Methods

### Primary tumours, tissues and cell lines

37 primary medulloblastoma samples were analysed in this study. The cohort included 22 male and 14 female patients (1 unknown), aged 1.3 to 40 years at diagnosis (1 infant (<3 years), 31 children (≥3 to 15 years) and 4 adults (≥16 years)). Histopathological review identified 27 cases of classic medulloblastoma, 4 cases of nodular/desmoplastic medulloblastoma, 5 cases of large-cell/anaplastic medulloblastoma and one case of undefined histological variant [12]. Four non-neoplastic cerebellar samples (aged 10 months to 67 years) were also investigated. All aspects of this study have been approved by the Newcastle & North Tyneside NHS Trust research ethics committee (approval 07/Q0905/71). Written Informed consent for tumour collection, storage and research was obtained for all primary medulloblastomas, from participants or their parents/legal guardians. Under the terms of the research ethics committee approval for this study, and the UK Human Tissue Act (2006), consent was not required for the normal cerebellar material, as it was collected prior to 2006. Eight medulloblastoma cell lines were investigated (DAOY, D384MED, D425MED, D458MED, D341MED, D283MED, MHH-MED-1, MHH-MED-8A), cultured and maintained as previously described (Langdon et al. 2006). Genomic DNA from samples was extracted using the Nucleon hard tissue kit (Nucleon Biosciences) or by the TRIzol method (Invitrogen), according to the manufacturer's instructions. RNA was extracted from samples using the TRIzol method (Invitrogen), and treated with DNAse I (Ambion) to eliminate contaminating DNA.

### SNP-array analysis

Genomic DNA samples from the eight medulloblastoma cell lines were analysed using the GeneChip® Mapping 10K 2.0 Array (Affymetrix), and raw data processed using Microsoft Excel spreadsheets, as previously described [13]. The raw SNP array data is available freely from the Cancer Genome Project, subject to completion of a data access agreement (http://www.sanger.ac.uk/genetics/CGP/Archive/). Data were used to identify markers displaying evidence of genomic amplification or homozygous deletion, which were defined as follows: intensity ratio (or haploid copy number (HCN))>2.5, amplification or high-level gain; HCN<0.1, homozygous deletion. Physical positions of probes and genes were identified according to the SNP consortium (TSC) database (http://www.hapmap.org/) and the NCBI database build 36.2 (http://www.ncbi.nih.nlm.gov). The estimated maximal regions of continuous defects affecting multiple probes were identified by the physical positions of the nearest flanking unaffected probe at each end.

### Quantitative real-time PCR

Analysis of hsa-miR-30b and hsa-miR-30d expression, measured relative to a U6 non-coding RNA control, was performed using proprietary TaqMan® MicroRNA real-time PCR Assay primer sets and reagents (Applied Biosystems), based on absolute quantification, following the manufacturer's instructions. All other analyses were performed using real-time PCR, based on the SYBR Green JumpStart Taq ReadyMix system (Sigma). Absolute quantification was applied to copy number analysis, whereas relative quantification was applied to expression analysis. *B2M*, *RPLP0* and *TBP* were used as reference genes for copy number analysis, and 28s rRNA was used as the reference gene for expression analysis. Details of primers used for these PCRs are shown in Supplementary Tables S1, S2 & S3. All real-time PCR assays were undertaken on an ABI PRISM 9700HT (Applied Biosystems) real-time PCR system.

### Fluorescence *in situ* hybridisation

Interphase fluorescence *in situ* hybridization (iFISH) was performed in medulloblastoma cell lines using BAC clones RP11-383N10 (representing sequences at 8q24.23; obtained from The Wellcome Trust Sanger Institute, Cambridge), dJ968N11 (representing *MYC* [6]) and pZ8.4 (representing the centromere of chromosome 8 [6]), as previously described [6], [14].

## Results

### SNP-array based copy number analysis of medulloblastoma cell lines: Identification of a novel region of recurrent genomic amplification at 8q23.22–q23.24 specific to medulloblastoma

**Figure 1:**
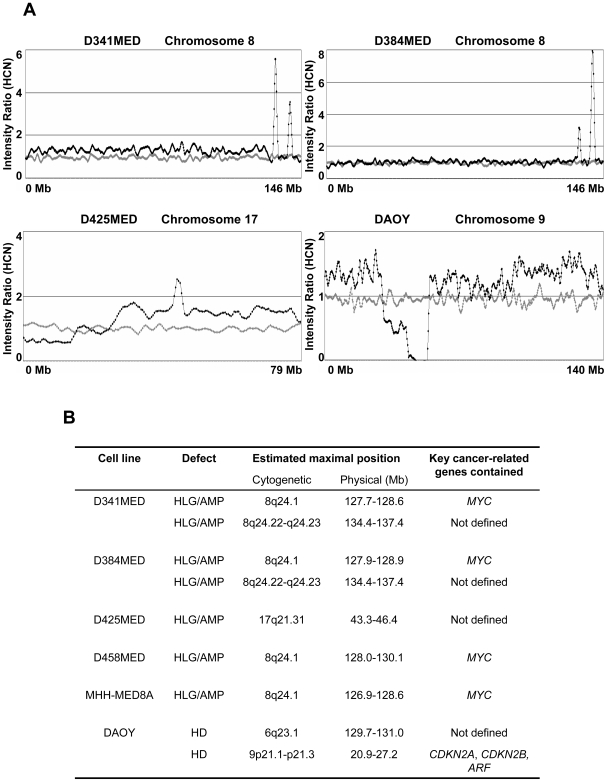
Homozygous deletions and genomic amplifications/high level gains identified in medulloblastoma cell lines. [A] Illustrative copy number studies for medulloblastoma cell lines showing defects detected by the SNP-array analysis. Data were analysed and displayed as previously described (Bignell, Huang et al. 2004). Probes are displayed by their physical position on the chromosome (X-axis), from p-ter to q-ter (left to right). The smoothed intensity ratio, based on a ‘moving window’ average of five adjacent probes is indicated by the Y-value. Mean values for a panel of 29 normal samples are represented by the grey dots, while the values of the medulloblastoma cell line are shown in black. In this figure, two independent amplifications/high level gains at 8q24 are shown in D341MED and D384MED, as well as an amplification/high level gain at 17q21 in D425MED and a homozygous deletion at 9p21 in DAOY. [B] Detailed information for defects identified affecting more than two adjacent markers. Estimated maximal cytogenetic and physical positions of each defect are shown, as well as any known key cancer-related gene contained within the region. HCN, estimated haploid copy number; HLG/AMP, high level gain or genomic amplification; HD, homozygous deletion.

Raw 10K SNP-array data generated from 8 medulloblastoma cell lines were analysed and converted to graphical plots, as previously described [13]. Wider chromosomal defects identified were consistent with our previous genome-wide studies of these cell lines using loss of heterozygosity and conventional comparative genomic hybridisation methods (data not shown [14]). However, high-resolution SNP array identified a number of focal defects for further investigation, and we elected to focus our analysis on these regions of putative genomic amplification or homozygous deletion, which may contain critical oncogenes or tumour suppressor genes in medulloblastoma development. Defects affecting at least three consecutive markers were classified as significant and, using these criteria, evidence of both genomic amplifications and homozygous deletions were observed (Figure 1A), which are summarised in Figure 1B. The majority of these regions either harboured known critical cancer-related genes or affected previously described features of medulloblastoma; amplification of *MYC* at 8q24.21, a well established feature of medulloblastoma [1], [2], [6], was the only recurrent event detected which affected an established cancer-related gene, seen in 4/8 cell lines analysed. Homozygous deletions of (i) the 9p21.1–p21.3 region encompassing *CDKN2A*, *CDKN2B* and *ARF* and (ii) the 6q23.1 region, each observed in a single cell line, have previously been reported in medulloblastoma and other cancer types [7], [8], [15], [16]. A novel high level gain/amplification at 17q21.31, in conjunction with evidence of isochromosome (17q) (i(17q)) (i.e. gain of the q-arm and loss of the p-arm of chromosome 17), the most common cytogenetic feature of medulloblastoma [1], [2], was found in the D425MED line.

Notably, a single novel region of recurrent genomic high level gain/amplification was observed, at 8q23.22–q23.24, in two independent medulloblastoma cell lines (D341MED and D384MED). This novel feature at 8q24.22–q24.23 is of particular interest since (i) it was observed in multiple cell lines, and (ii) no evidence of amplification at this region was found in the analysis of over 800 other cancer cell lines derived from a wide range of different paediatric and adult cancer types using the GeneChip® Mapping 10K 2.0 Array (http://www.sanger.ac.uk/cgi-bin/genetics/CGP/cghviewer/CghHome.cgi), suggesting this putative amplicon may contain gene(s) specifically related to medulloblastoma development. Additionally, we confirmed the independence of the D341MED and D384MED cell lines; each displayed a unique allelotype and harboured distinct genomic defects [14]. Therefore, we elected to further characterise this novel region in detail, and its role in medulloblastoma.

### Verification, characterisation and physical mapping of the novel amplification at 8q24.22–q24.23 in medulloblastoma cell lines

**Figure 2:**
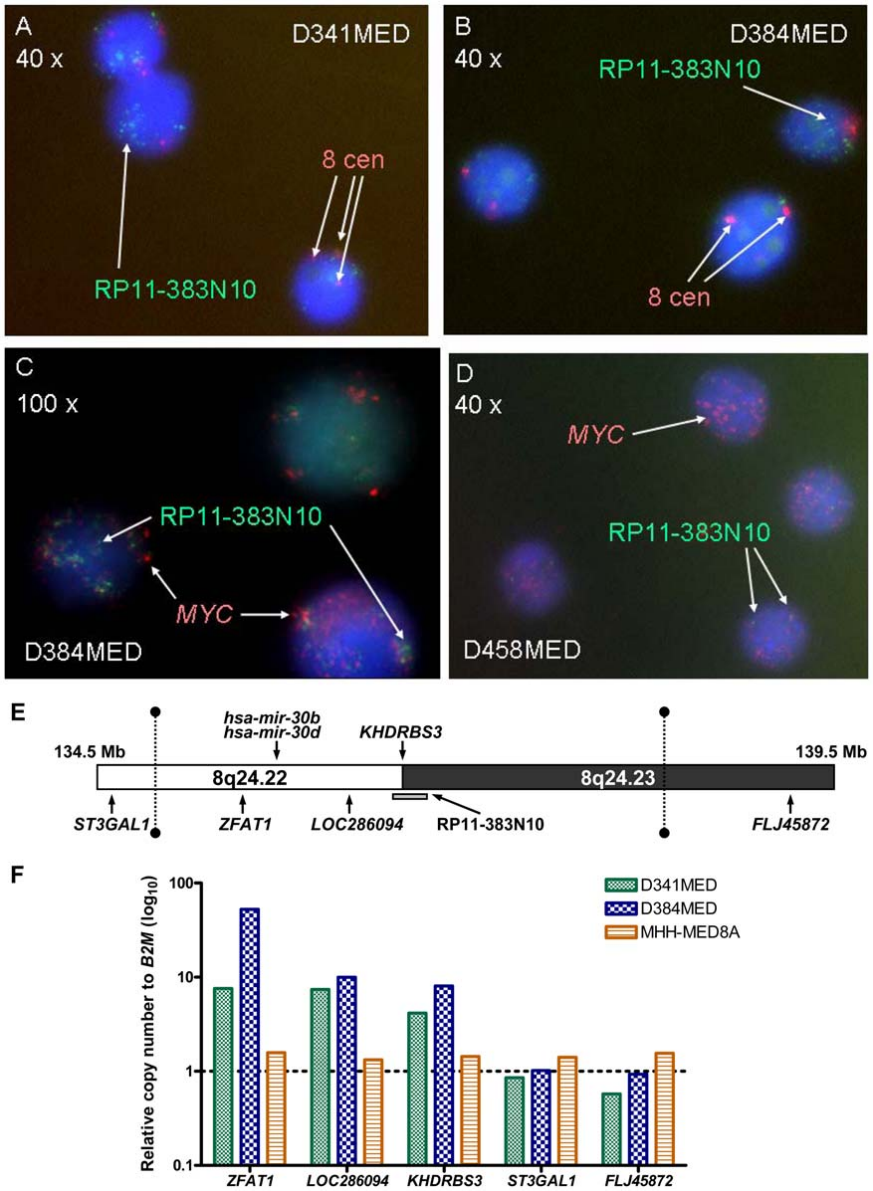
Verification and physical mapping of the novel amplification at 8q24.22–q24.23 in medulloblastoma cell lines. iFISH analysis using the RP11-383N10 and chromosome 8 centromeric probes in [A] D341MED and [B] D384MED. Red signals represent the chromosome 8 centromeric probes, and the green signals represent the RP11-383N10 probes. iFISH analyses using the RP11-383N10 and *MYC* probes are shown in [C] D384MED and [D] D458MED. Red signals represent the *MYC* probes, and the green signals represent the RP11-383N10 probes. Nuclei were counter-stained using DAPI (blue) in all experiments. Magnifications are indicated. [E] A physical map of the novel amplicon at 8q24.22–q24.23 identified in medulloblastoma cell lines. The putative minimal common region of amplification identified by SNP array analysis is indicated by the two vertical dotted lines with solid ends. The position of the BAC-clone used for FISH analysis is indicated by the grey bar. [F] Real-time PCR analysis of the relative copy number status of genes located within and around the novel amplicon at 8q24.22–q24.23 in the medulloblastoma cell lines D341MED, D384MED and MHH-MED8A. The relative copy number of each candidate gene is shown relative to the reference gene, *B2M*, and is shown as a log_10_ scale. Two replicate analyses of each sample were performed and the mean value is shown.

The novel high-level gain/amplification observed at 8q24.22–q24.23 in D341MED and D384MED cell lines by SNP array was next investigated independently by iFISH. The BAC-clone RP11-383N10, which represents DNA sequences within this amplicon (Figure 2E), was used as a probe, and confirmed the presence of DNA amplification (>10 copies per control centromeric signal) in both cell lines (Figures 2A & B), but not in the other cell lines examined (data not shown).

We noted that amplification of *MYC* at 8q24.21 and the novel region at 8q24.22–q24.23 was co-incident in both D341MED and D384MED. Although SNP-array data indicate that the regions of amplification are discrete (Figure 1A), the relationship between this novel amplification and *MYC* amplification at 8q24.21, was assessed by co-hybridization of RP11-383N10 and BAC probe dJ968N11, recognising *MYC*, in D384MED cells, to investigate whether their amplification was co-localised on a single amplicon. The combined result from over 100 cells indicated a mixed pattern of amplification in the cell population; *MYC* amplification alone was observed in 32% of cells, while the novel amplification alone was observed in 28% of the cell population. 22% of the cells showed amplification of both regions, but in two thirds of these cells, signals generated by the two BAC probes were not co-localised (Figure 2C).

The amplification status and physical gene content of the novel amplicon was additionally investigated using real-time PCR-based mapping. Analysis of the status of the coding genes within and surrounding the putative amplicon (Figure 2E) showed that *ZFAT1*, *LOC286094* and *KHDRBS3* were amplified in both cell lines D341MED and D384MED, but not in the non-amplified MHH-MED-8A line. The flanking genes, *ST3GAL1* and *FLJ45872*, at the proximal and distal ends respectively of the amplified region, did not shown any evidence of amplification in any cell line (Figure 2F). *LOC100130092*, which encodes a predicted psudogene within the amplified region on the NCBI database (http://www.ncbi.nlm.nih.gov/), was not investigated. By SNP-array, physical mapping identified the amplicon extending from 134,810,164 bp (tsc0062042) to 137,820,222 bp (tsc0696464) in D341MED, and from 134,810,164 bp (tsc0062042) to 137,858,383 bp (tsc1392944) in D384MED, indicating a maximal common region of 3 Mb, extending from 134,810,164 bp to 137,820,222 bp.

Together, these data indicate that the novel amplification at 8q24.22–q24.23 is independent of *MYC* amplification and has consecutive gene content in both D341MED and D384MED, with the involved regions extending from *ZFAT1* at its proximal end to *KHDRBS3* at its distal end, and also encompasses *LOC286094* and two genes encoding micro-RNAs (mi-RNAs), hsa-miR-30b and hsa-miR-30d.

### Identification of putative target gene(s) of the novel amplification at 8q24.22–q24.23: investigation of relationships between gene amplification and transcript levels

**Figure 3:**
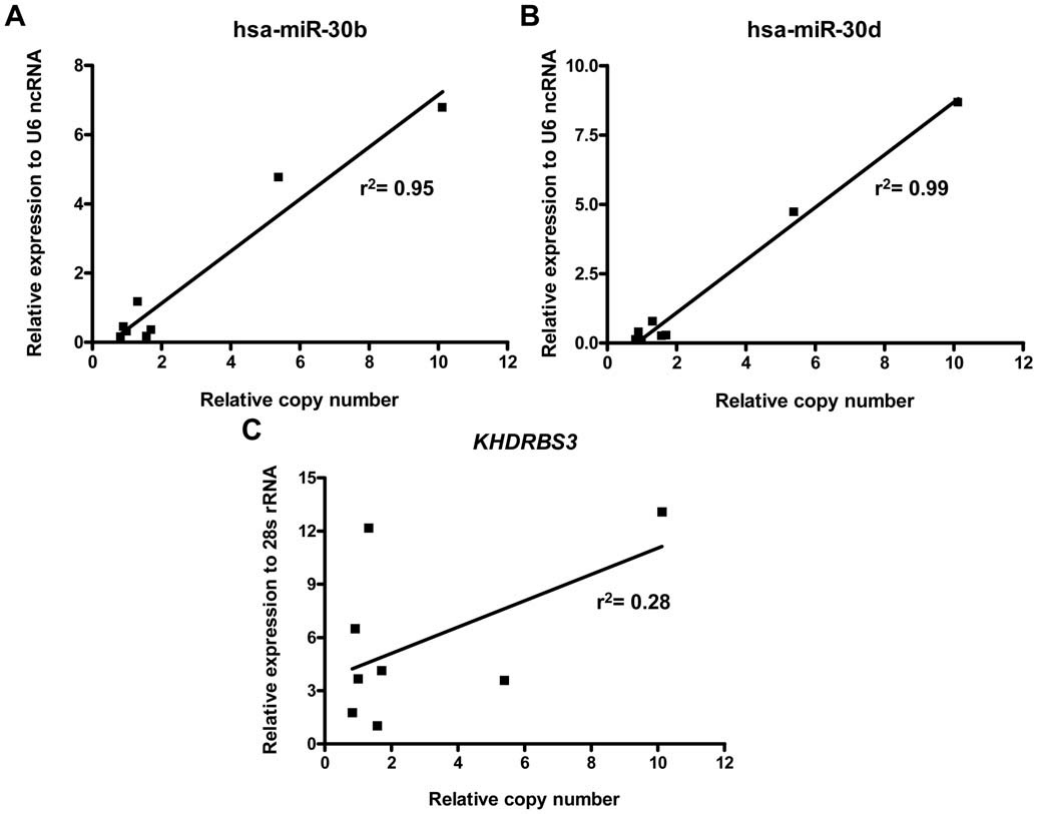
Association between transcript level and copy number of genes located in the novel amplicon at 8q24.22–q24.23 in medulloblastoma cell lines. [A] hsa-miR-30b [B] hsa-miR-30d [C] *KHDRBS3*. Linear regression analyses were performed and the r^2^ values are indicated.

To identify potential target genes of the novel amplification which may contribute to tumourigenesis, relationships between the transcript levels and copy numbers of genes contained in the novel amplicon were assessed in medulloblastoma cell lines using real-time PCR methods. High-level expression of hsa-miR-30b and hsa-miR-30d was closely correlated with their copy number status (Figures 3A & B), suggesting these may represent targets. No evidence of high-level expression of *ZFAT1* or *LOC286094*, or correlation with gene copy number, was found in any cell line (data not shown), suggesting these do not represent candidate target genes. Data obtained for *KHDRBS3* were less clear; high-level expression was seen in three cell lines, however only one cell line of the three harboured the amplification, while no evidence of high-level expression was observed in the other amplified cell line (Figure 3C). Based on these findings, we next investigated the expression of the two mi-RNAs and *KHDRBS3* in primary medulloblastomas and normal cerebellum, to further explore any roles in medulloblastoma development.

### Transcript levels of candidate amplicon target genes and miRNAs in primary medulloblastomas and the normal cerebellum

**Figure 4:**
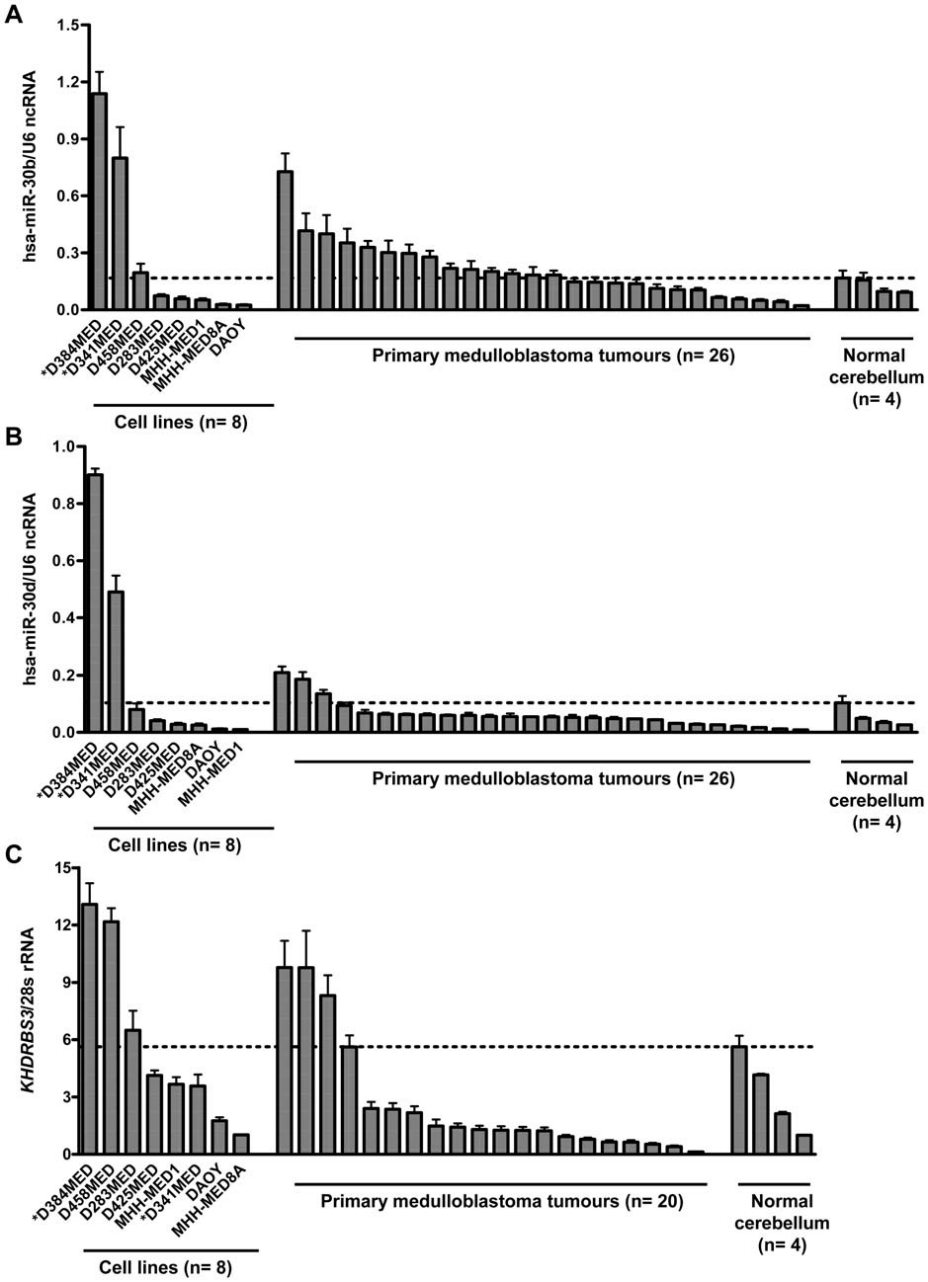
Transcript levels of hsa-miR-30b, hsa-miR-30d and *KHDRBS3* in primary medulloblastoma tumours and the normal cerebellum. Expression of each candidate relative to its reference gene is indicated in the Y-axis. The samples were grouped into three categories (X-axis): medulloblastoma cell lines, primary medulloblastoma tumours and normal non-neoplastic cerebellum, and are shown from left to right in order from the highest expression level to the lowest in each group. Three replicate analyses were performed for each sample. Mean results and standard errors (represented by the error bars) are shown. The threshold for overexpression detection is indicated by the dotted line. * indicates the two cell lines harbouring the novel amplification at 8q24.22–q24.23.

Expression of mature hsa-miR-30b and hsa-miR-30d, as well as *KHDRBS3*, was analysed in cohorts of primary medulloblastoma samples, alongside four normal cerebellar samples, and data for medulloblastoma cell lines (n = 8) (Figure 4). The highest mean level of expression observed in a normal cerebellar tissue sample was used as a threshold for the determination of overexpression in primary tumours and cell lines. Using these criteria, 54% (14/26), 12% (3/26) and 15% (3/20) of primary medulloblastomas showed evidence of overexpression of hsa-miR-30b, hsa-miR-30d, and *KHDRBS3*, respectively (Figure 4). High-level expression of hsa-miR-30b, comparable to levels observed in 8q24.22–q24.23 amplified cell lines, was observed in a sub-set of primary tumours. High-level expression of *KHDRBS3*, equivalent to levels observed in an 8q24.22–q24.23 amplified cell line, was also observed in a sub-set of primary tumours and other cell lines, but was not restricted to the 8q24.22–q24.23 amplified cell lines. Although expression levels of hsa-miR-30d in primary tumours were elevated relative to the normal cerebellum, they did not approach levels observed in 8q24.22–q24.23 amplified cell lines.

### Analysis of genetic amplification at 8q24.22–q24.23 in primary medulloblastomas

The relative copy number of *KHDRBS3*, as a marker of the novel amplicon at 8q24.22–q24.23, was determined in all primary medulloblastoma samples in which the expression level was assessed (n = 37) by qPCR; no evidence of genomic amplification was observed (data not shown).

## Discussion

This genome-wide SNP-array based screen has facilitated the characterisation of a series of focal regions of gene amplification and homozygous deletion in medulloblastoma, and the identification of putative target genes which may play critical roles in its development, and have therapeutic significance. In addition to the identification and further validation of established genomic events in medulloblastoma (e.g. *MYC* amplification, *CDKN2A/B* homozygous deletion [1], [2], [6]–[8]), defects have been identified which will, following further validation, aid investigations of the critical genetic targets of common medulloblastoma chromosomal aberrations. i(17q) is the most common chromosomal defect in medulloblastoma, affecting 30–40% of cases, and chromosome 6 loss is characteristic of the molecular disease sub-group defined by activation of the Wnt/Wingless cell signalling pathway [1]–[5]. The respective high level gain/amplification identified at 17q21.31, and the homozygous deletion at 6q23.1, will yield candidate target genes and facilitate the further mapping and investigation of these chromosomal aberrations.

The novel region of recurrent amplification identified at 8q24.33–q24.23 formed the focus of our further investigations. In addition to its recurrent nature, we have demonstrated its high-copy number amplification, independence of *MYC* amplification, and uniqueness to medulloblastoma amongst a panel of >800 cell lines derived from different tumour types, indicating this novel amplicon contains key gene(s) in medulloblastoma development, with the potential to inform critical insights to disease pathogenesis. The identification of the genetic target(s) of this novel amplicon, and their validation in studies of primary tumours, and in functional models, is therefore now paramount. Our detailed genetic mapping of its physical location and gene content, and investigation of their relationship to gene expression in medulloblastoma cell lines, identified *KHSRBS3* and the genes encoding mi-RNA species, hsa-miR-30b and hsa-miR-30d, as putative oncogenic targets whose expression is closely correlated with amplification status.

In primary tumours, although we did not detect evidence of amplification of this target region in our cohort of primary medulloblastomas, consistent with data from a recent SNP-array screen of 212 primary medulloblastomas [8], our investigations of the transcript levels of these target species further support roles in medulloblastoma. Based on these data, all three candidates, *KHSRBS3*, hsa-miR-30b and hsa-miR-30d, could be considered as potential targets of this novel amplification, as all showed elevated expression in a sub-set of primary medulloblastomas, measured relative to the normal cerebellum. hsa-miR-30b is arguably of most interest because, in addition to its high-level expression in cell lines which is closely correlated with amplification, it showed the greatest frequency of elevated expression in primary tumours (over 50% of cases), with expression levels detected in certain cases equivalent to those observed in amplified cell lines. Although such investigations were beyond the scope of the limited initial primary tumour cohort employed in the current study, the clinico-pathological correlates of expression of all candidates in extensive, well characterised, and statistically powered clinical cohorts, is additionally likely to inform any potential roles in medulloblastoma.

Importantly, none of the three candidates can be fully excluded at this stage, and further investigations of their functional roles in medulloblastoma are required. The function(s) of *KHDRBS3*, hsa-miR-30b and hsa-miR-30d, and in particular any roles they may play in tumourigenesis, have not been widely investigated, and this is the first study, to our knowledge, to investigate their status in medulloblastoma. KHDRBS3 (KH domain containing, RNA binding, signal transduction associated 3) belongs to a RNA binding protein family, and regulates the variant splicing of certain target genes [17]. Although one published study has demonstrated that expression of KHDRBS3 positively regulates telomerase activity in human colon cancer HCT-116 cells [18], further details of *KHDRBS3* function and association with cancers are not clear. Similarly, hsa-miR-30b and hsa-miR-30d have not been well characterised. Array-based studies have confirmed their expression in human tissues [19]–[21], however on-line databases (e.g. miRBase; http://microrna.sanger.ac.uk/) predict >800 mRNA targets for each species, and their specific functions remain unclear. Nonetheless, the amplification and overexpression of hsa-miR-30b and hsa-miR-30d in medulloblastoma further supports an emerging role for the dysregulation of mi-RNA species in medulloblastoma development [22]–[26].

In summary, we have described a genome-wide screen of medulloblastoma cell lines to identify and characterise regions of gene amplification and homozygous deletion which may harbour critical genes in its development. Our findings implicate hsa-miR-30b, hsa-miR-30d and *KHDRBS3* as putative oncogenic target(s) of a novel recurrent medulloblastoma amplicon at 8q24.22–q24.23, which is independent of *MYC* amplification and unique to this disease. These data suggest specific and critical roles for these genes in medulloblastoma development for wider investigation, and further support the contribution of mi-RNA species to medulloblastoma pathogenesis.

## Supporting Information

Table S1 Primers for real-time PCR analysis of the genomic copy number status of genes contained within the novel amplicon at 8q24.22–q24.23. All sequences are shown in the 5′ to 3′ direction. F-forward, R-reverse. The predicted length of each PCR product is shown in base pairs (bp). The annealing temperature for all PCRs shown was 60°C.
(0.04 MB DOC)

Table S2 Primers for real-time PCR analysis of transcript expression of genes contained within the novel amplicon at 8q24.22–q24.23. All sequences are shown in the 5′ to 3′ direction. F-forward, R-reverse. The predicted length of each PCR product is shown in base pairs (bp). The annealing temperature for all PCRs shown was 60°C. Primers recognising ZFAT1 and KHDRBS3 genes were designed to span two or more exons, to control against contamination by genomic DNA sequences.
(0.03 MB DOC)

Table S3 Primers for real-time PCR analysis of copy number status of KHDRBS3 in primary medulloblastoma samples. All sequences are shown in the 5′ to 3′ direction. F-forward, R-reverse. The putative length of each PCR product is shown in base pairs (bp). The annealing temperature for all PCRs shown was 60°C.
(0.03 MB DOC)
