# Supplementary material for: Amplification and Overexpression of Hsa-miR-30b, Hsa-miR-30d and KHDRBS3 at 8q24.22-q24.23 in Medulloblastoma
Source: PLoS One. 2009 Jul 7;4(7):e6159. doi: 10.1371/journal.pone.0006159 (PMC2702821; doi:10.1371/journal.pone.0006159)
Supplement: Table S1 — Primers for real-time PCR analysis of the genomic copy number status of genes contained within the novel amplicon at 8q24.22–q24.23. All sequences are shown in the 5′ to 3′ direction. F-forward, R-reverse. The predicted length of each PCR product is shown in base pairs (bp). The annealing temperature for all PCRs shown was 60°C. (0.04 MB DOC) [file pone.0006159.s001.doc]

| **Name** |  | **Sequence** | **Length (bp)** |
| --- | --- | --- | --- |
| *ST3GAL1* | F | CTGCCCAATCACCTGTCCAC | 81 |
|  | R | GAGGGCGTAAGAGGGACTGG |  |
|  |  |  |  |
| *ZFAT1* | F | TTGCTGGAGTCCAAGTGACCT | 81 |
|  | R | TGAACTGGTAGGCTCGGAAGA |  |
|  |  |  |  |
| *LOC286094* | F | GTGGCGTGCATAGGGTGC | 81 |
|  | R | AGGCCGTGGATGAGTTTGAC |  |
|  |  |  |  |
| *KHDRBS3* | F | CAAGTAGAGCGAAGAGCATTAG | 124 |
|  | R | GATGGCATTAAACACCCTAGA |  |
|  |  |  |  |
| *FLJ45872* | F | AATTCACCCAACAGGCCTCC | 101 |
|  | R | TCCAGTCCTAGCACGTTGTGC |  |
|  |  |  |  |
| *B2M* | F | TCTAGGCGCCCGCTAAGTT | 81 |
|  | R | TCGCGTGCTGTTTCCTCC |  |
