# Supplementary material for: Amplification and Overexpression of Hsa-miR-30b, Hsa-miR-30d and KHDRBS3 at 8q24.22-q24.23 in Medulloblastoma
Source: PLoS One. 2009 Jul 7;4(7):e6159. doi: 10.1371/journal.pone.0006159 (PMC2702821; doi:10.1371/journal.pone.0006159)
Supplement: Table S2 — Primers for real-time PCR analysis of transcript expression of genes contained within the novel amplicon at 8q24.22–q24.23. All sequences are shown in the 5′ to 3′ direction. F-forward, R-reverse. The predicted length of each PCR product is shown in base pairs (bp). The annealing temperature for all PCRs shown was 60°C. Primers recognising ZFAT1 and KHDRBS3 genes were designed to span two or more exons, to control against contamination by genomic DNA sequences. (0.03 MB DOC) [file pone.0006159.s002.doc]

| **Name** |  | **Sequence** | **Length (bp)** |
| --- | --- | --- | --- |
| *ZFAT1* | F | TGCTGTCGGAAGTTCTCCAA | 100 |
|  | R | TCAGACTCGTTACCTGCTTCTCCT | exon 3-4 |
|  |  |  |  |
| *KHDRBS3* | F | CCAGCCCAAAGTGGTGCT | 121 |
|  | R | CTGTCCTCGCTGAAGGTGC | exon 7-9 |
|  |  |  |  |
| *28S rRNA* | F | TTGAAAATCCGGGGGAGAG | 100 |
|  | R | ACATTGTTCCAACATGCCAG |  |
