# Supplementary material for: Amplification and Overexpression of Hsa-miR-30b, Hsa-miR-30d and KHDRBS3 at 8q24.22-q24.23 in Medulloblastoma
Source: PLoS One. 2009 Jul 7;4(7):e6159. doi: 10.1371/journal.pone.0006159 (PMC2702821; doi:10.1371/journal.pone.0006159)
Supplement: Table S3 — Primers for real-time PCR analysis of copy number status of KHDRBS3 in primary medulloblastoma samples. All sequences are shown in the 5′ to 3′ direction. F-forward, R-reverse. The putative length of each PCR product is shown in base pairs (bp). The annealing temperature for all PCRs shown was 60°C. (0.03 MB DOC) [file pone.0006159.s003.doc]

| **Name** |  | **Sequence** | **Length (bp)** |
| --- | --- | --- | --- |
| *B2M* | F | TCTAGGCGCCCGCTAAGTT | 81 |
|  | R | TCGCGTGCTGTTTCCTCC |  |
|  |  |  |  |
| *RPLP0* | F | CTTTTAGTGGCCATGGATCTGC | 81 |
|  | R | AGCTGCACATCACTCTGAACCA |  |
|  |  |  |  |
| *TBP* | F | GAAATAGGGTAAAGTGATAGGAGGACA | 75 |
|  | R | AGGCCGACTTGTAGTCGTCAGT |  |
|  |  |  |  |
| *KHDRBS3* | F | CAAGTAGAGCGAAGAGCATTAG | 124 |
|  | R | GATGGCATTAAACACCCTAGA |  |
